# Trimethylamine-N-Oxide Impedes Late Endothelial Progenitor Cell–Mediated Revascularization by Triggering Mitochondrial Apoptosis via Suppression of MnSOD

**DOI:** 10.1155/cdr/9910333

**Published:** 2025-06-18

**Authors:** Yijia Shao, Jiapan Sun, Xiang Liu, Xing Liu, Fang Wu, Zhichao Wang, Shiyue Xu, Long Chen

**Affiliations:** ^1^Department of Geriatrics, The First Affiliated Hospital, Sun Yat-sen University, Guangzhou, China; ^2^Department of Traditional Chinese Medicine, The Seventh Affiliated Hospital, Sun Yat-sen University, Shenzhen, China; ^3^Department of Cardiac Surgery, Guangdong Cardiovascular Institute, Guangdong Provincial People's Hospital (Guangdong Academy of Medical Sciences), Southern Medical University, Guangzhou, China; ^4^Department of Cardiology, The Third Affiliated Hospital, Sun Yat-sen University, Guangzhou, China; ^5^The International Medical Department of Shenzhen Hospital, Southern Medical University, Shenzhen, China; ^6^Department of Hypertension and Vascular Diseases, The First Affiliated Hospital, Sun Yat-sen University, Guangzhou, China; ^7^National Clinical Research Center of Cardiovascular Diseases, Fuwai Hospital Chinese Academy of Medical Sciences, Shenzhen, China

## Abstract

**Background and Aims:** Trimethylamine-N-oxide (TMAO) is recognized as a novel marker and mediator of atherosclerotic cardiovascular disease (ASCVD). Endothelial progenitor cells (EPCs) are crucial for maintaining vascular homeostasis. Impaired EPC numbers and function correlate with increased adverse cardiovascular events. The aim of this study was to decipher the effect of TMAO on late EPCs (LEPCs) and its underlying molecular mechanism.

**Methods and Results:** In vitro migration and tubulogenic capacities of LEPCs were attenuated by TMAO in a dose-dependent manner, accompanied by inhibition of manganese superoxide dismutase (MnSOD) and mitochondrial damage. TMAO-induced mitochondrial damage provoked proinflammatory responses (increased levels of IL-6, IL-1b, ICAM-1, E-sel, and TNF-*α*) and autophagic cell death (confirmed by western blot immunofluorescent staining and transmission electron microscopy) in LEPCs. Overexpression of MnSOD through adenovirus transfection reversed TMAO-related LEPCs dysfunction. To study the effect of TMAO on LEPC-mediated vascular repair in vivo, a hind limb ischemia model was established in nude mice, and LEPCs were injected in the ischemic hind limb. Laser Doppler imaging of mouse ischemic hindlimbs at 21 days indicated that TMAO treatment inhibited LEPCs-mediated blood flow recovery, which was restored by MnSOD overexpression. Immunohistology analyses further revealed consistent alterations in capillary density determined by CD31 staining.

**Conclusions:** TMAO induces mitochondrial damage in LEPCs via MnSOD suppression, which leads to cell dysfunction, proinflammatory activation, and autophagic cell death in vitro and impaired LEPCs-mediated revascularization in vivo. Overexpression of MnSOD restores TMAO-induced LEPCs dysfunction and further enhances LEPC-mediated revascularization in the ischemic hind limbs in nude mice.

## 1. Introduction

Trimethylamine-N-oxide (TMAO) is a metabolite that originates from bacterial metabolism of choline-rich foods, such as red meats and eggs, in the large intestine and is rapidly oxidized by flavin-containing Monooxygenase-3 in the liver [1, 2]. Increased plasma TMAO level is considered an independent predictor of adverse cardiovascular events in both coronary artery disease (CAD) patients and the general population [3–5]. TMAO has been reported to contribute to endothelial dysfunction, vascular inflammation, platelet hyperreactivity, and the development of atherosclerosis [6–8].

Endothelial progenitor cells (EPCs) are critical for maintaining the integrity and function of vascular endothelium and the revascularization of ischemic tissue [9–12]. The number of circulating EPCs is inversely correlated with cardiovascular risk factors and has declined in CAD patients [13, 14]. Of note, a recent study showed that plasma TMAO levels were negatively correlated with the number of circulating EPCs and flow-mediated vasodilatation (FMD) [15]. However, the detailed mechanisms underlying TMAO-related EPC dysfunction remain unclear. Emerging evidence indicated that TMAO induced endothelial dysfunction via oxidative stress [6, 16]. LEPCs express intrinsically high levels of MnSOD, and decreased MnSOD in LEPCs is a critical contributor to the impaired capacity of angiogenesis [17–19]. Hence, we hypothesized that TMAO provoked mitochondrial oxidative stress in LEPCs by downregulating MnSOD, which led to cell dysfunction and consequently attenuates LEPC-mediated revascularization in the ischemic tissues.

In this study, we sought to decipher the role of TMAO in the regulation of LEPC function. Our results showed that TMAO induced mitochondrial oxidative stress, which triggered the proinflammatory activation as well as autophagic cell death in LEPCs by suppressing MnSOD expression. Upregulation of MnSOD restored TMAO-induced LEPC dysfunction in vitro and enhanced LEPC-mediated reparative effect in hind limb ischemic mice. Collectively, our study demonstrates a crucial role of MnSOD in TMAO-mediated regulation of LEPC function and provides a new perspective for the therapeutic intervention for cardiovascular diseases.

## 2. Material and Methods

### 2.1. Ethical Statement

Twenty healthy subjects aged 18–55 years were enrolled in the study. All subjects gave their written informed consent before study entry. Exclusion criteria included malignancy, cardiovascular events, active inflammatory disease, and those with other cardiovascular risk factors or taking other medications. The ethics approval was granted by the Ethics Committee of the First Affiliated Hospital, Sun Yat-sen University in Guangzhou ([2017] 154), China.

Male BALB/c nude mice (6–8 weeks) were purchased from the Experimental Animal Center of Sun Yat-sen University, used for animal experiment, and kept under controlled environmental conditions (constant laminar airflow, 20°C–23°C, 40%–60% relative humidity, and 12/12 h light/dark cycle). The animal experiments were reviewed and approved by the committee review of animal experiments in the First Affiliated Hospital of Sun Yat-sen University ([2021] 049). All animal protocols followed the Guide for the Care and Use of Laboratory Animals published by the US National Institutes of Health (National Institutes of Health Publication No. 85-23, revised 1996).

### 2.2. LEPC Culture, Identification, and Treatment

LEPCs were cultured as previously described [20]. Briefly, peripheral blood mononuclear cells from healthy subjects were isolated using Ficoll separation medium by density gradient centrifugation and then cultured in EBM-2 medium (endothelial cell growth basal medium; Lonza) supplemented with 20% fetal bovine serum (Sigma-Aldrich), 10 ng/mL VEGF (vascular endothelial growth factor), 4 ng/mL FGF (fibroblast growth factor), and 10 ng/mL EGF (epidermal growth factor; US Biologicals) and antibiotics (100 U/mL penicillin and 100 *μ*g/mL streptomycin). After 21–28 days of culture, the adherent mononuclear cells dually positive for endothelial markers including CD31, CD309, CD34 and CD45 (BD Pharmingen) were identified as LEPCs by flow cytometry (Cytoflex, Beckman Coulter, United States) as previously described.

TMAO was purchased from Sigma-Aldrich, Missouri, United States. It was dissolved in PBS. LEPCs were treated with different concentrations of TMAO (100, 200, 400, and 800 *μ*mol/L) for 24 h. To monitor the occurrence of autophagy flux, LEPCs were treated with 100 nmol/L bafilomycin A1 (MedChemExpress, New Jersey, United States) for 2 h following the addition of TMAO (400 *μ*mol/L) for another 24 h.

### 2.3. Cell Viability Measurements

The Cell Counting Kit-8 (CCK-8) (Dojindo, Kumamoto, Japan) was utilized to assess the viability of LEPCs exposed to TMAO, according to the manufacturer's instructions. Briefly, LEPCs were seeded into a 96-well microplate (Corning Life Sciences, Corning, New York) at a density of 1 × 10^4^ LEPCs per well and exposed to varying concentrations of TMAO for 24 h (100, 200, 400, 800 *μ*mol/L) or a constant concentration (400 *μ*mol/L) at different time intervals (12, 24, 48, and 72 h). Subsequently, 10 *μ*L of CCK-8 solution was added to each well, followed by incubation at 37°C for 2 h. Viable cells were quantified via absorbance measurements using a monochromator microplate reader (Tecan Group Ltd, Mannedorf, Switzerland) at a wavelength of 450 nm. The optical density at 450 nm was evaluated to determine the percentage of cell viability relative to the control group, which was set at 100%.

### 2.4. In Vitro Migration and Scratch Assay

The migration ability of LEPCs was evaluated using a modified Boyden chamber (Costar Transwell assay, 8 *μ*m pore size, Corning, New York). The chamber was placed in a well containing 500 *μ*L EBM-2 supplemented with PBS in a 24-well culture plate. 2 × 10^4^ LEPCs were resuspended in 250 *μ*L EBM-2 medium and pipetted into the upper chamber. After incubation at 37°C for 24 h, transmigrated cells were fixed with 4% paraformaldehyde for 15 min and 0.3% crystal violet for another 15 min for further enumeration and analysis.

A “scratch” was created by scraping the culture cell monolayer in a straight line with a p200 pipet tip. The cells were washed with culture medium for three times to remove the debris and then cultured for 24 h. The scratch healing was recorded by photographing under an inverted microscope (10 × magnification) and analyzed using ImageJ software.

### 2.5. In Vitro Tube Formation

Growth factor reduced Matrigel (Corning) was warmed up at 4°C overnight, and 100 *μ*L of Matrigel was plated in 48-well plates and incubated for 1 h at 37°C. 8 × 10^5^ LEPCs were resuspended in EBM-2 medium, loaded on top of the Matrigel, and then incubated at 37°C. The tube formation was imaged directly under a microscope at several time points. An average of tubules was counted from 3 to 5 random fields.

### 2.6. Adenoviral Transduction

To evaluate the various stages of autophagy, treated LEPCs were transduced with adenovirus vector LC3 (Ad-mRFP-GFP-LC3) which were purchased from HanBio Technology (Shanghai, China) at a multiplicity of infection of 200 for 8 h, and the medium was replaced with fresh growth medium 48 h after infection. Images were obtained by the LSM780 laser scanning confocal microscope (ZEISS, Germany) system.

LEPCs were transduced with adenovirus encoding the human SOD2 gene (Ad-SOD2) or the negative control gene (Ad-NC) (Genechem company, Ltd., Shanghai, China) at a multiplicity of infection of 200, and the medium was replaced with fresh growth medium 48 h after infection (the specific results were shown in Figure S1).

### 2.7. Mitochondrial Membrane Potential (MMP) Disruption

MMP disruption was detected with JC-1 cationic dye (5 *μ*g/mL, KeyGen Biotech, China), respectively. Briefly, LEPCs were labeled with one of the specific fluorescent dyes for 30 min at 37°C. Then, cells were washed and resuspended with PBS at a concentration of 1 × 10^6^ cells/mL. The percentage of labeled cells was analyzed via a FACS Canto flow cytometry (BD Biosciences) and LSM-710 confocal microscopy imaging (Carl Zeiss).

### 2.8. Autophagosome and Autolysosome Ultrastructure Imaging

Autophagosome and autolysosome ultrastructure of LEPCs was imaged by transmission electron microscopy (FEI Tecnai G^2^ Spirit Twin, United States). LEPCs were fixed with a buffer containing 2.5% glutaraldehyde for 24 h and refixed in 1% osmium tetroxide for 30 min. After that, cells were dehydrated in graded ethanol and washed with propylene oxide before being embedded and sectioned.

### 2.9. Detection of Apoptosis

The apoptosis rate of LEPCs was detected with Annexin V-FITC/propidium iodide (PI) apoptosis detection kit (BD Biosciences). According to the manufacturer's instructions, treated LEPCs were washed with cold PBS and binding buffer. Then, cells were resuspended in binding buffer and incubated with Annexin V-FITC and PI for 15 min in the dark at room temperature. Finally, LEPCs were added to binding buffer for dilution and analyzed by flow cytometry (Cytoflex, Beckman Coulter, United States).

### 2.10. Real-Time PCR Analysis

Total RNA was extracted with Trizol reagent (Invitrogen, California, United States), and cDNA was synthesized using the PrimeScript RT reagent kit (Takara Biotechnology, Japan). The mRNA expression of GAPDH, IL-6, IL-1b, ICAM1, E-Selectin, and TNF-*α* was quantified using the 2^−ΔΔCT^ analytical method in triplicate using a Step One Plus real-time PCR System (ABI, United States). The mRNA level of the GAPDH gene was measured in each sample as an internal normalization standard. The primer GAPDH (sense:5⁣′-TGCACCACCAACTGCTTAGC⁣′, antisense:5⁣′-GGCATGGACTGTGGTCATGAG-3⁣′), IL-6 (sense:5⁣′-TTCAATGAGGAGACTTGCCTG-3⁣′,antisense:5⁣′CTGGCATTTGTGGTTGGGTC-3⁣′), IL-1b (sense:5⁣′-ACAACTGCACTACAGGCTCC-3⁣′, antisense:5⁣′-TGTCGTTGCTTGGTTCTCCT-3⁣′), ICAM1 (sense:5⁣′-ACGGAGCCAATTTCTCATGC-3⁣′,antisense:5⁣′- TTGGGATGGTAGCTGGAAGATC-3⁣′), E-Selectin (sense: 5⁣′- ccccgaagggtttggtgagg-3⁣′, antisense: 5⁣′- ccggaactgccaggcttgaa-3⁣′), and TNF-*α* (sense:5⁣′-GTACCTTGTCTACTCCCAGGTTCTCT -3⁣′, antisense: 5⁣′-GTGTGGGTGAGGAGCACGTA -3⁣′) were synthesized by Ruibiotech (Beijing, China). Real-time PCR was performed in a 20-*μ*L reaction mixture containing primers, FastStart Universal SYBR Green master (ROX) reagent (Roche Applied Science, Mannheim, Germany) and 2-*μ*L cDNA sample.

### 2.11. Western Blot Analysis

Total protein was extracted and quantified by cytoBuster TM protein extraction reagent (Beyotime Biotechnology, China) and bicinchoninic acid protein assay kit (Thermo, United States) separately. Protein extracts were subjected to SDS-PAGE, transferred to polyvinylidene fluoride membranes (Roche, Indianapolis, Indiana, United States). The following antibodies were used: mouse anti-MnSOD antibody (1:500; Santa cruz, United States), anti-Bcl-2 antibody (1:1000; Immunoway, United States), rabbit anti-Bax antibody (1:1000; Immunoway, United States), and rabbit anti-GADPH antibody (1:1000; Cell Signaling Technology) rabbit anti-ACTB antibody. Proteins were visualized with HRP-conjugated anti-rabbit IgG (1:2000; Cell Signaling Technology), followed by use of the ECL chemiluminescence system (Thermo). The original uncut western blot membranes were shown in Figure S2.

### 2.12. Hind Limb Ischemia Model

The hindlimb ischemia models were performed as described in our previous study [20]. Briefly, mice were anesthetized with 2.5% isoflurane and maintained with 1.5% isoflurane and placed in dorsal recumbency with hind limbs externally rotated. A skin incision was made over the femoral artery, beginning at the inguinal ligament and continuing caudally to the popliteal bifurcation. The femoral artery was isolated above the level of the profunda and epigastric arterial branches, doubly ligated using 7–0 Prolene suture, and transected. The SFA caudal to the major branch points was dissected, ligated, and excised in its entirety. LEPCs (1 × 10^5^ cells) were resuspended in 100 *μ*L of prewarmed PBS (37°C) and injected intramuscularly into three sites of ischemic gastrocnemius muscle. The same volume of PBS was injected into control mice. Hind limb subcutaneous blood flow was detected using a Laser Doppler imager (Perimed Instruments, Sweden) at 0, 3, 7, 14, and 21 days after surgery. After 21 days, mice were euthanized through cervical dislocation under anesthesia, and hind limb muscle tissues were harvested for further analyses.

### 2.13. Immunohistochemical Staining

The muscle tissues were harvested rapidly, washed with normal saline, fixed in 4% formaldehyde, and embedded in paraffin. The paraffin blocks were cut into 2-*μ*m thick sections. The muscle tissues were deparaffinized and rehydrated, and antigen retrieval was carried outby microwave oven heating in sodium citrate buffer (0.01 mol/L, pH 6.0). Sections were incubated with rabbit monoclonal CD31 antibody (Cell Signaling Technology Inc., Massachusetts, United States) followed by HRP Anti-Rabbit DAB Detection kit (Cell Signaling Technology Inc., Massachusetts, United States) according to the manufacturers' instructions. Images were acquired using a light microscope (Olympus BX63) with 400x magnification.

### 2.14. Statistical Analysis

All results were expressed as the mean ± SEM and plotted using GraphPad Prism 8 software. Two groups of normal distribution data were analyzed using 2-tailed Student's *t*-test. Statistical significance of multiple groups was assessed by one-way analysis of variance (ANOVA) followed by Tukey's test. Biological experiment replicates in each group were specified in the figure legends (*N* value). *p* < 0.05 was considered to denote statistical significance. All statistical analyses were performed using SPSS statistical software (SPSS Version 23.0).

## 3. Results

### 3.1. Characterization of Late EPC

**Figure 1:**
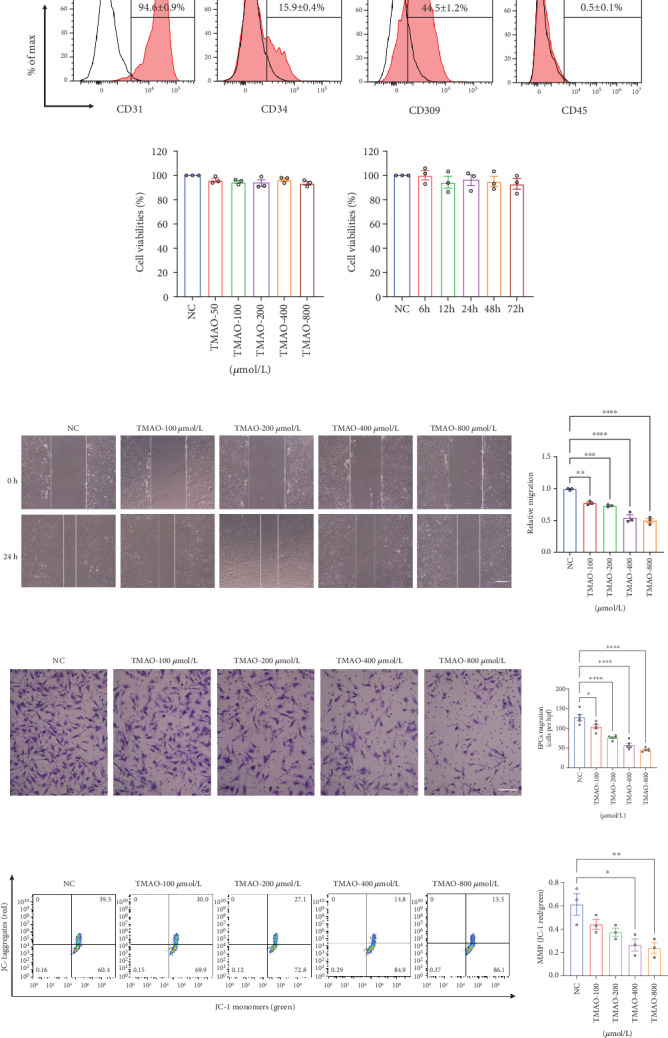
TMAO impairs LEPC function and induces mitochondrial damage in a dose-dependent manner. (a) Representative flow cytometry analyses of EC markers (CD31, CD309, and CD34) and monocyte marker CD45 in LEPCs. (b) Quantification of CCK-8 cytotoxicity assay. (c) Representative images and quantification of scratch-wound assay to determine migration area over 24 h. Scale bar: 100 *μ*m (*n* = 3). (d) Representative images and quantification of transwell assay of migration capacity. Scale bar: 100 *μ*m (*n* = 5). (e) Representative and quantification of flow cytometry analyses of JC-1 staining to determine mitochondrial membrane potential (*n* = 3) (Mean ± SEM; ⁣^∗^*p* < 0.05, ⁣^∗∗^*p* < 0.01, ⁣^∗∗∗^*p* < 0.001, and ⁣^∗∗∗∗^*p* < 0.0001 vs. NC).

After 21–28 days of culture, the LEPCs were identified using flow cytometry. The expression levels of three endothelial cell markers were CD31 (94.6 ± 0.9%), CD309 (44.5 ± 1.2%), and CD34 (15.9 ± 0.4%), while the monocyte marker CD45 was 0.5 ± 0.1% (Figure 1). These cells were subsequently used in the following experiments.

### 3.2. TMAO Dose-Dependently Hampered LEPC Function and Provoked Mitochondrial Damage In Vitro

The CCK-8 cytotoxicity assay was employed to assess the cytotoxicity of TMAO in LEPCs. LEPCs were exposed to varying concentrations of TMAO (100, 200, 400, and 800 *μ*mol/L) for 24 h or to a fixed concentration of TMAO (400 *μ*mol/L) across different time intervals (12, 24, 48, and 72 h). As shown in Figure 1, TMAO exerted no significant cytotoxic effect on LEPCs. To investigate the effect of TMAO on LEPC function, LEPCs were treated with PBS control and TMAO at different doses (100, 200, 400, and 800 *μ*mol/L) for 24 h. LEPC migration capacity was tested with scratch-wound experiments and transwell assays. As shown in Figure 1, LEPCs treated with TMAO demonstrated impaired migration ability in a dose-dependent manner both in scratch-wound experiments and transwell assays compared with PBS-treated ones. To evaluate mitochondrial damage, we assessed MMP disruption with JC-1 cationic dye in LEPCs treated with or without TMAO treatment. TMAO treatment significantly increased MMP disruption in a dose-dependent manner compared with the PBS control group (Figure 1). These results indicated that TMAO might induce LEPC dysfunction via mitochondrial damage.

### 3.3. TMAO Activated Proinflammatory Responses and Autophagic Cell Death in LEPCs

**Figure 2:**
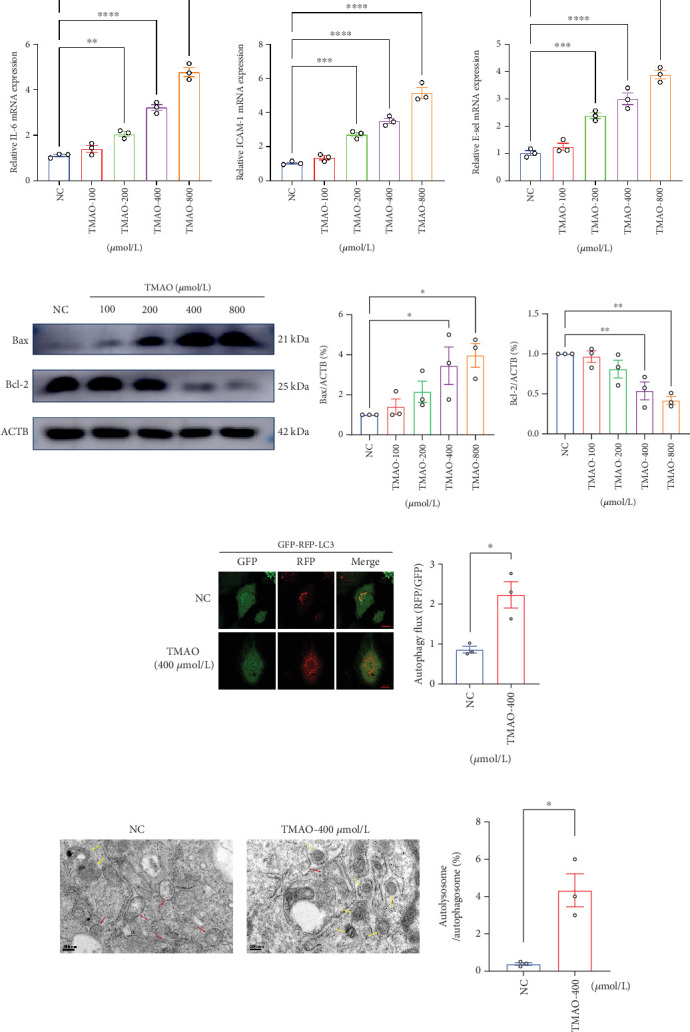
TMAO provokes the proinflammatory response, autophagy, and apoptosis in LEPCs. (a) RT-PCR analyses of proinflammatory cytokines mRNA levels in LEPCs treated with or without TMAO (*n* = 3). (b) Western Blotting analyses of Bcl-2 and Bax levels in LEPCs treated with or without TMAO. (c) Autophagy flux was detected by confocal microscopy in LEPCs transfected with Ad-mRFP-GFP-LC3 (GFP indicates autophagosome; mRFP indicates autolysosome. Scale bar: 20 *μ*m; *n* = 3). (d) Autophagic vacuoles and autolysosomes were quantified by transmission electron microscopy; red arrows denote autophagosome, and yellow arrows indicate autolysosomes. Scale bar: 0.2 *μ*m (Mean ± SEM; ⁣^∗^*p* < 0.05, ⁣^∗∗^*p* < 0.01, ⁣^∗∗∗^*p* < 0.001, and ⁣^∗∗∗∗^*p* < 0.0001 vs. NC).

Given that mitochondrial damage is a potent trigger of inflammation and cell apoptosis, we further studied the effect of TMAO on inflammatory activation and cell apoptosis in LEPCs. The mRNA expression levels of proinflammatory factors (IL-6, ICAM-1, and E-selectin) were analyzed in LEPCs treated with PBS or TMAO for 24 h. In comparison with the PBS treated group, LEPCs treated with TMAO exhibited markedly increased levels of IL-6, ICAM-1, and E-selectin in a dose-dependent manner (Figure 2a). Then, we measured the protein level of Bax and Bcl-2 in LEPCs. Consistent with the increased mitochondrial damage, TMAO treatment significantly promoted the expression of Bax and inhibited the Bcl-2 level, which indicated a shift to proapoptotic status (Figure 2b). More interestingly, we observed enhanced cell autophagic activities in LEPCs treated with TMAO. As shown in Figure 2c,d, both immunofluorescent staining and transmission electron microscopy results demonstrated an increased number of autolysosomes in the TMAO treated group compared to the PBS control group. Taking together, our results suggest that TMAO induced mitochondrial damage may lead to inflammatory activation and autophagic cell death in LEPCs.

### 3.4. MnSOD Mediated the Mitochondrial Damage and Dysfunction in LEPCs Induced by TMAO

**Figure 3:**
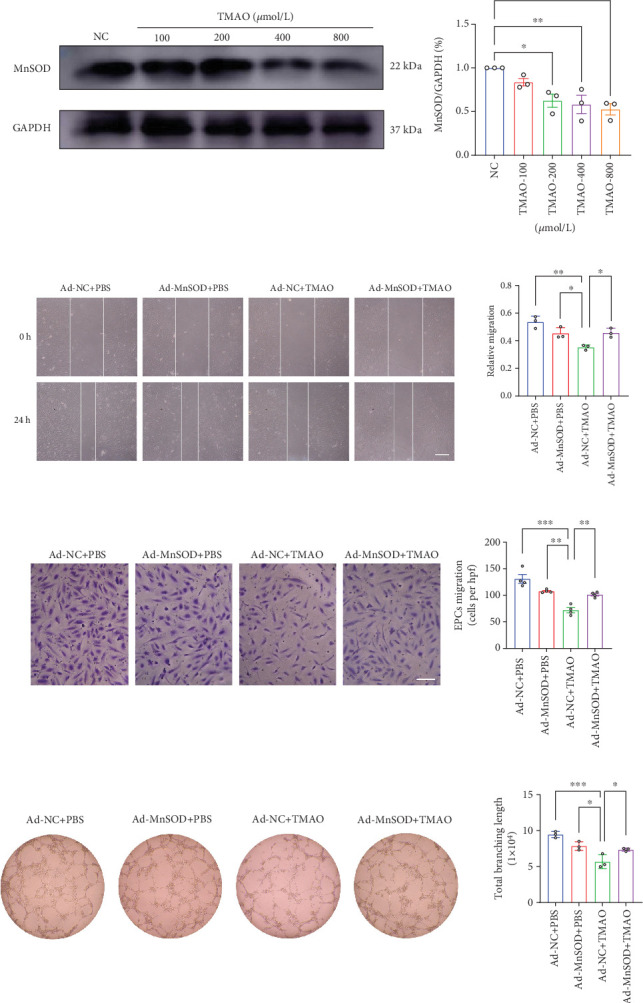
TMAO-induced functional deterioration of LEPCs by inhibiting MnSOD. (a) Western Blotting analyses of MnSOD expression in LEPCs treated with or without TMAO. (*n* = 3, ⁣^∗^*p* < 0.05 and ⁣^∗∗^*p* < 0.01 vs. NC). (b) Representative images and quantification of scratch-wound assay to determine migration area over 24 h. Scale bar: 100 *μ*m (*n* = 3, ⁣^∗∗^*p* < 0.01 vs. Ad-NC + PBS, ⁣^∗^*p* < 0.05 vs. Ad-NC + TMAO). (c) Representative images and quantification of transwell assay of migration capacity. Scale bar: 100 *μ*m (*n* = 4, ⁣^∗∗∗^*p* < 0.001 vs. Ad-NC + PBS, ⁣^∗∗^*p* < 0.01 vs. Ad-NC + TMAO). (d) Representative images and quantification of matrigel tube formation assay over 6 h in LEPCs. Scale bar: 50 *μ*m (*n* = 3, ⁣^∗∗∗^*p* < 0.001 vs. Ad-NC + PBS, ⁣^∗^*p* < 0.05 vs. Ad-NC + TMAO) (Mean ± SEM).

To further investigate the molecular mechanism underlying TMAO-induced mitochondrial injury and cell dysfunction in LEPCs, we analyzed the level of MnSOD, an essential mitochondrial antioxidant enzyme that detoxifies the free radical superoxide, in LEPCs treated with TMAO. Our results displayed that TMAO suppressed the protein expression of MnSOD (Figure 3a). Moreover, overexpression of MnSOD through adenovirus transfection (Ad-MnSOD) remarkably restored TMAO-induced impaired cell migration (Figure 3b,c) and tube-forming (Figure 3d) capacities of LEPCs compared with the nontarget control (Ad-NC).

**Figure 4:**
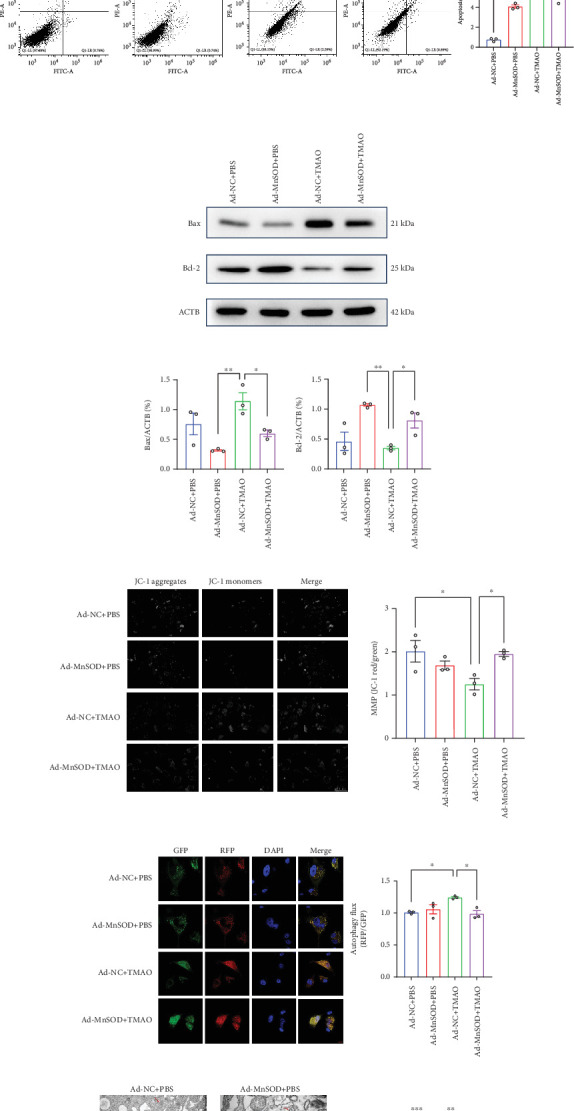
MnSOD overexpression reverses TMAO-induced proinflammatory response and mitochondrial apoptosis in LEPCs. (a) RT-PCR analyses of proinflammatory cytokines mRNA levels in LEPCs (*n* = 3, ⁣^∗∗∗^*p* < 0.001 vs. Ad-NC + TMAO). (b) Representative images and quantification of low cytometry analyses of cell apoptosis in LEPCs (*n* = 3, ⁣^∗∗∗^*p* < 0.001 vs. Ad-NC + PBS, ⁣^∗^*p* < 0.05 vs. Ad-NC + TMAO). (c) Western Blotting analyses of Bcl-2 and Bax level in LEPCs (*n* = 3, ⁣^∗∗∗^*p* < 0.001 vs. Ad-NC + PBS, ⁣^∗∗^*p* < 0.01 vs. Ad-NC + TMAO; ⁣^∗^*p* < 0.05 vs. Ad-MnSOD+TMAO). (d) Representative and quantification of JC-1staining to determine mitochondrial membrane potential. Scale bar: 50 *μ*m (*n* = 3, ⁣^∗^*p* < 0.05 vs. Ad-NC + TMAO). (e) Autophagy flux was detected by confocal microscopy in LEPCs transfected with Ad-mRFP-GFP-LC3 (GFP indicates autophagosome; mRFP indicates autolysosome. Scale bar: 0.5 *μ*m. *n* = 3, ⁣^∗^*p* < 0.05 vs. Ad-NC + TMAO). (f) Autophagic vacuoles and autolysosomes were quantified by transmission electron microscopy (red arrows denote autophagosome; yellow arrows indicate autolysosomes. Scale bar: 0.5 *μ*m. *n* = 3, ⁣^∗∗∗^*p* < 0.001 vs. Ad-NC + TMAO; ⁣^∗∗^*p* < 0.01 vs. Ad-MnSOD+TMAO) (Mean ± SEM).

In addition, the mRNA levels of proinflammatory factors (IL-6, IL-1b, ICAM-1, and TNF-*α*) in Ad-MnSOD LEPCs were significantly lower than Ad-NC LEPCs after TMAO treatment (Figure 4a). Moreover, MnSOD-overexpression reversed cell apoptosis and the mitochondrial damage induced by TMAO treatment in LEPCs (Figures 4b, 4c, and 4d). The autophagy activity was also attenuated in Ad-MnSOD LEPCs compared with the Ad-NC group after TMAO treatment (Figure 4e,f)). Overall, these results demonstrated that MnSOD played a key role in TMAO-regulated mitochondrial damage and consequent cell dysfunction and apoptosis in LEPCs.

### 3.5. TMAO Impeded LEPC-Mediated Revascularization in Hind Limb Ischemic Mice by Suppressing MnSOD

**Figure 5:**
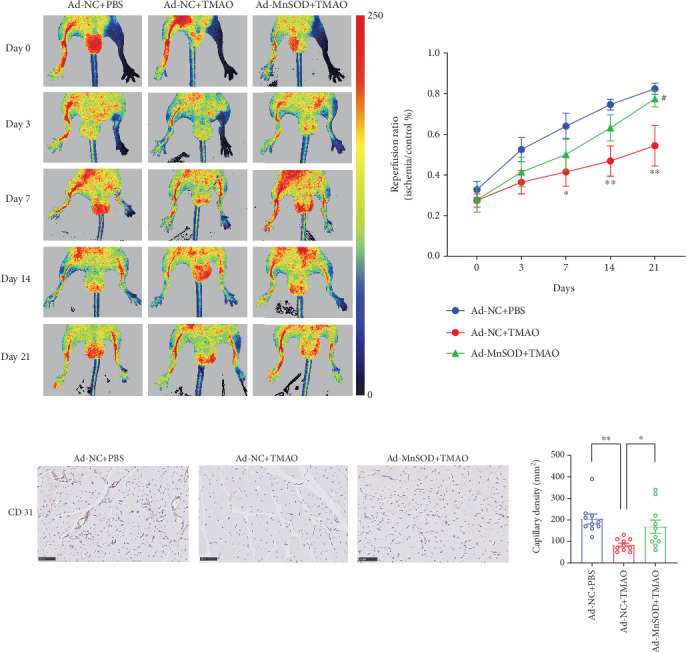
MnSOD overexpression restores TMAO-induced impaired reparative capacity of LEPCs in vivo. (a) Reperfusion assessed by laser Doppler imaging at 21 days postligation with blood flow quantified as a ratio in ischemic versus healthy control limb (*n* = 5, ⁣^∗^*p* < 0.05 and ⁣^∗∗^*p* < 0.01 vs. Ad-NC + PBS, #*p* < 0.05 vs. Ad-NC + TMAO). (b) Capillary density was measured by immunohistochemical staining (*n* = 10, ⁣^∗∗^*p* < 0.01 vs. Ad-NC + PBS, ⁣^∗^*p* < 0.05 vs. Ad-MnSOD+TMAO) (Mean ± SEM).

Considered the in vitro data clearly implicating MnSOD as a key regulator of TMAO induced LEPCs dysfunction, we next determined the in vivo functionality of the TMAO/MnSOD pathway in a mouse model of hind limb ischemia. Notably, the rescue of blood perfusion at 21 days was attenuated in the Ad-NC + TMAO group compared to the Ad-NC + PBS group, while MnSOD overexpression markedly reversed the impaired reperfusion ratio (Ad-MnSOD+TMAO vs. Ad-NC + TMAO) (Figure 5a).

In order to more specifically assess the effects of TMAO/MnSOD on revascularization in the ischemic limb, we performed immunohistochemical staining of CD31. CD31 positive vascular-like structures were clearly elevated in the Ad-MnSOD+TMAO group compared with the Ad-NC + TMAO group, which is consistent with improved blood reperfusion (Figure 5b). Taken together, these data indicate that TMAO remarkably attenuated LEPC-mediated revascularization in vivo, which could be restored by MnSOD overexpression.

## 4. Discussion

Here, we demonstrated for the first time that TMAO suppressed MnSOD expression and induced mitochondrial damage in LEPCs, which led to proinflammatory activation and autophagic cell death in vitro and impaired LEPCs-mediated revascularization in hind limb ischemic mice. MnSOD overexpression in LEPCs restored TMAO-induced impaired function in vitro and the reparative effect in vivo. Our data provide novel insight into the mechanisms underlying TMAO-induced LEPC dysfunction and expand our understanding of the relationship between TMAO and ASCVD.

The importance of gut microbiota, as a novel endocrine organ, in the regulation of cardiovascular function has been recognized in the past decade [21, 22]. Emerging evidence indicated that the level of TMAO, a metabolite derived from the gut microbiota, is closely associated with endothelial dysfunction and the increased risk of major adverse cardiovascular events [1, 3]. In the presented study, we confirmed that TMAO treatment deteriorated the migration and tube-forming abilities in a dose-dependent manner. Moreover, TMAO-treated LEPCs exhibited enhanced expression of proinflammatory factors, including IL-6, IL-1b, ICAM-1, E-Sel, and TNF-*α*, and activated autophagic cell death. Although Chou et al. [15] recently reported that increased plasma TMAO levels in patients with stable angina were associated with reduced circulating EPC numbers and impaired function. Our results suggest that TMAO could cause extensive injury and provoke autophagy-mediated cell apoptosis in LEPCs. Given the important role of LEPCs in maintaining the homeostasis of vascular function, TMAO-induced LEPC malfunction and apoptosis may contribute to the increased risk of adverse cardiovascular events in ASCVD patients with high plasma TMAO.

Mitochondrial damage is an essential trigger of proinflammation activation and cell apoptosis [23, 24]. Intriguingly, recent studies indicated that TMAO impaired endothelial cell function via mitochondrial damage [6, 25]. It has been reported that TMAO-induced NLRP3 activation occurred through the inhibition of SOD2 and increased production of mitochondrial reactive oxygen species [26]. To determine whether mitochondrial damage was involved in TMAO-induced LEPC dysfunction and apoptosis, we assessed the MMP disruption level in LEPCs. We found that TMAO dose-dependently augmented MMP disruption in LEPCs. Of note, in contrast with the increased MMP disruption, MnSOD level was remarkably suppressed by TMAO. Ample evidence suggests the key role of MnSOD in the clearance of mtROS and maintenance of LEPC function [19, 27]. Deficiency of MnSOD is highly associated with impaired LEPC-mediated vascularization [17, 28, 29]. To confirm the effect of MnSOD on LEPCs treated with TMAO, we overexpressed MnSOD via adenovirus transfection. Notably, overexpression of MnSOD in LEPCs treated with TMAO significantly attenuated mitochondrial damage and restored the impaired LEPC function, as well as ameliorated proinflammatory activation and autophagic cell death. Hence, our results indicated that MnSOD was the essential mediator of TMAO-related LEPC deterioration.

EPCs are capable of postnatal vasculogenesis and enhance tissue repair after ischemic vascular injury [10, 30, 31]. Revascularization mediated by autologous patient-derived EPC is a promising therapeutic strategy for enhancing vascular repair in ischemic diseases [32, 33]. Thus, we further explored the effect of TMAO on LEPCs-mediated revascularization in vivo in hind limb ischemic nude mice. Our results showed that TMAO treatment hindered the reparative capacities of LEPCs, which can be reversed by MnSOD overexpression. The decline in the number and function of EPCs is considered to be biomarkers for ASCVD. Our results provide evidence that impaired EPC function may be involved in the TMAO-associated adverse cardiovascular outcomes in ASCVD patients. These findings have significant implications for therapeutic strategies aimed at vascular repair in patients with ASCVD by restoring TMAO-mediated impaired LEPC functions.

## 5. Limitations and Perspectives

This study possesses several limitations. First, while our data suggest that MnSOD could serve as a therapeutic target to enhance the reparative capacity of LEPCs, this hypothesis necessitates further clinical investigation. Moreover, additional research is imperative to elucidate the upstream regulatory mechanisms through which TMAO modulates MnSOD activity and LEPC function. Finally, apart from mitochondrial damage mediated by MnSOD suppression, the roles of endoplasmic reticulum–mitochondrial calcium signaling and inflammatory pathways in TMAO's effects on LEPC function warrant further investigation.

## 6. Conclusion

**Figure 6:**
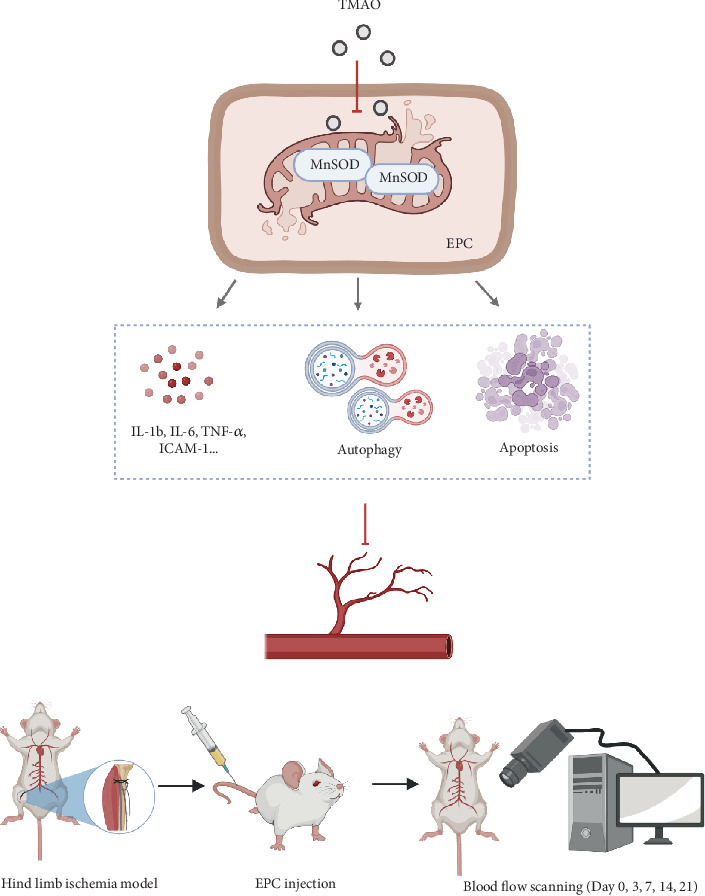
Schematic illustration. (a) Trimethylamine-N-oxide triggers mitochondrial apoptosis of LEPCs via suppression of MnSOD. (b) The experimental flow of revascularization in hind limb ischemic mice.

In summary, our data reveals that TMAO impairs LEPC function by suppressing MnSOD expression and provoking mitochondrial damage as illustrated in Figure 6. This study provides insight into the mechanism whereby TMAO affects LEPC-mediated vascular repair. Taken together, our results indicate that MnSOD may be a potential therapeutic target for improving LEPCs reparative effect in ASCVD patients with high TMAO levels.

## Data Availability

The data that support the findings of this study are available from the corresponding authors upon reasonable request.

## Supporting Information

Supporting Information Additional supporting information can be found online in the Supporting Information section. Figure S1. Titration of virus transfection efficiency. Figure S2. The uncut western blot.
