# Supplementary material for: Trimethylamine-N-Oxide Impedes Late Endothelial Progenitor Cell–Mediated Revascularization by Triggering Mitochondrial Apoptosis via Suppression of MnSOD
Source: Cardiovasc Ther. 2025 Jun 18;2025:9910333. doi: 10.1155/cdr/9910333 (PMC12197513; doi:10.1155/cdr/9910333)
Supplement: Supporting Information — Additional supporting information can be found online in the Supporting Information section. Figure S1. Titration of virus transfection efficiency. Figure S2. The uncut western blot. [file 9910333.f1.docx]

**Supplemental Material**

**Trimethylamine-N-Oxide impedes late endothelial progenitor cells mediated revascularization by triggering mitochondrial apoptosis via suppression of MnSOD**

Authors: Yijia Shao^1†^, Jiapan Sun^4†^, Xiang Liu^5†^, Xing Liu^6^, Fang Wu^1^, Zhichao Wang^2^, Shiyue Xu^3*^, Long Chen^2*^

^1^Department of Geriatrics, The First Affiliated Hospital, Sun Yat-sen University, Guangzhou, China

^2^The International Medical Department of Shenzhen Hospital, Southern Medical University, Shenzhen, China

^3^Department of Hypertension and Vascular Diseases, The First Affiliated Hospital, Sun Yat-sen University, Guangzhou, China

^4^Department of Traditional Chinese Medicine, The Seventh Affiliated Hospital, Sun Yat‑Sen University, Shenzhen, China

^5^Department of Cardiac Surgery, Guangdong Cardiovascular Institute, Guangdong Provincial People’s Hospital, Guangdong Academy of Medical Sciences, Guangzhou, China

^6^Department of Cardiology, The Third Affiliated Hospital, Sun Yat-sen University, Guangzhou, China

†These authors have contributed equally to this work and share first authorship.

*co-Corresponding author.

Long Chen

Email: chenl_sz@smu.edu.cn

Full address: The International Medical Department of Shenzhen Hospital, Southern Medical University, Shenzhen, 518000, China

Shiyue Xu

Email: xsy279@163.com

Full address: Department of Hypertension and Vascular Diseases, The First Affiliated Hospital, Sun Yat-sen University, Guangzhou 510080, China

**Supplementary Figure S1**


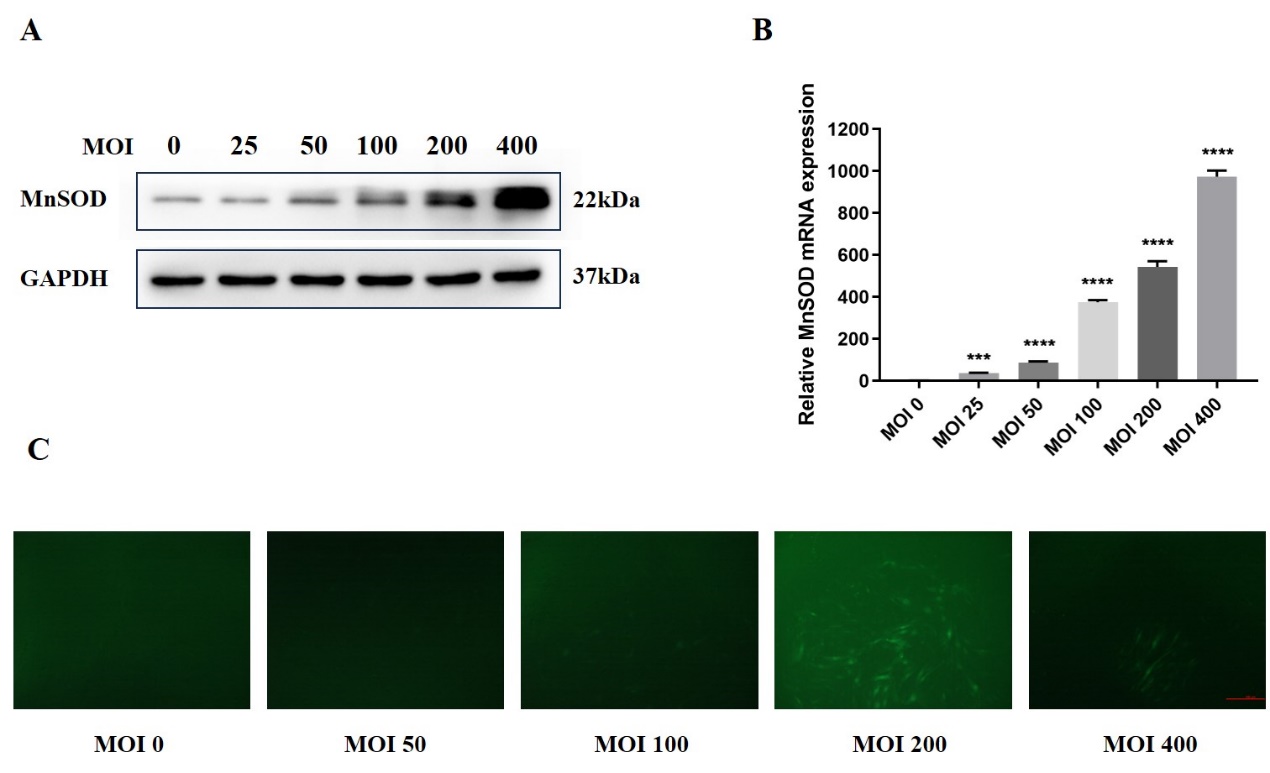


**Figure S1 Titration of virus transfection efficiency**

(A) Representative Western Blot of MnSOD expression level in LEPCs infected with multiple MOIs

(B) RT-PCR analyses of MnSOD mRNA levels in LEPCs infected with multiple MOIs.

(n=3, ***P <0.001 and ****P<0.0001 vs. MOI 0, Mean ± SEM).

(C) Representative fluorescence microscope image of LEPCs infected with multiple MOIs.

**Supplementary Figure S2**


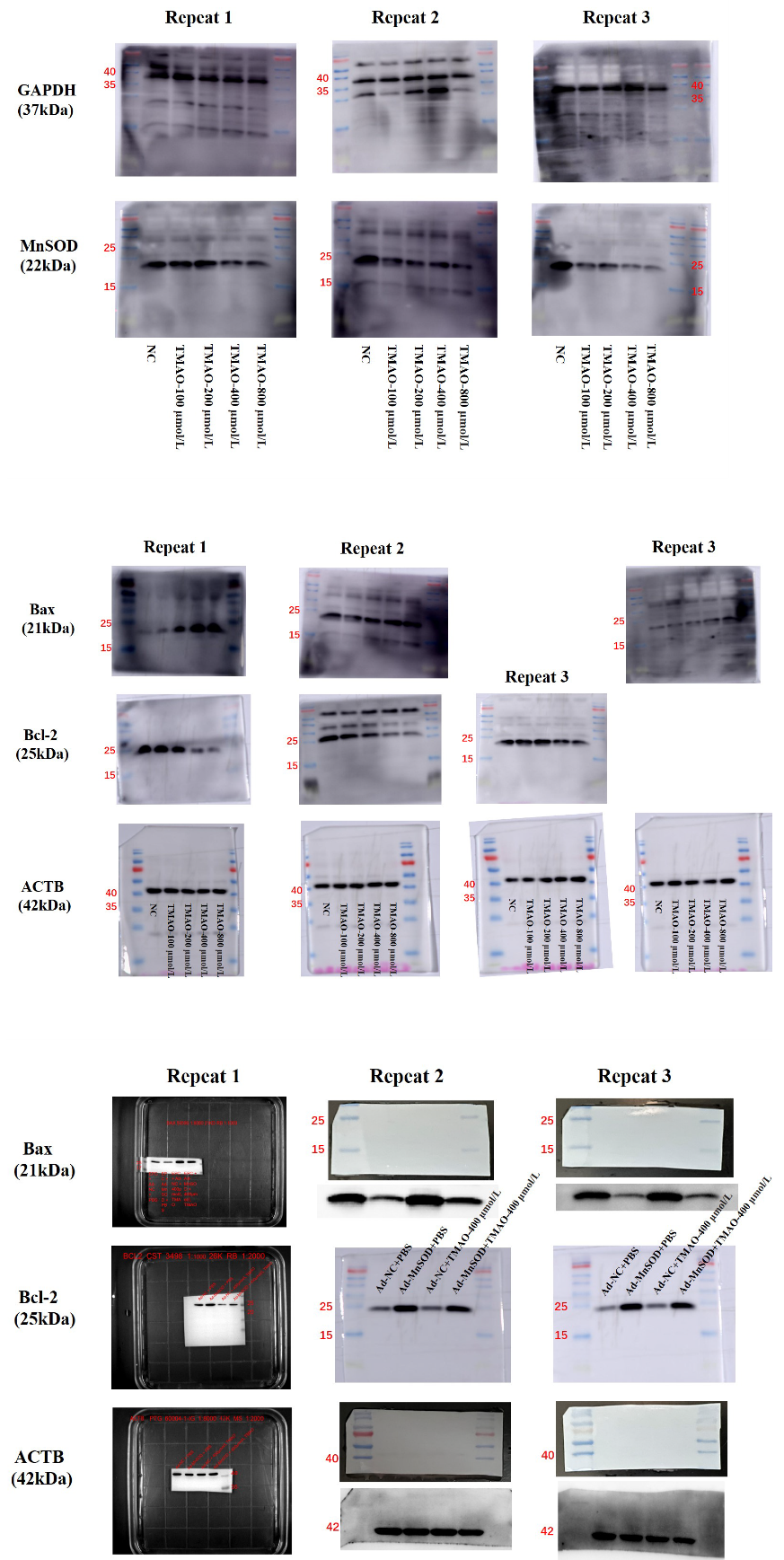


**Figure S2. The uncut western blot**

The original uncut western blot membranes for three repeats.
